# Regulation of *Rac1* transcription by histone and DNA methylation in diabetic retinopathy

**DOI:** 10.1038/s41598-021-93420-4

**Published:** 2021-07-08

**Authors:** Renu A. Kowluru, Rakesh Radhakrishnan, Ghulam Mohammad

**Affiliations:** Ophthalmology, Visual and Anatomical Sciences, Kresge Eye Institute, Wayne State University, Detroit, MI 48201 USA

**Keywords:** Diseases, Endocrinology

## Abstract

Cytosolic ROS, generated by NADPH oxidase 2 (Nox2) in diabetes, damage retinal mitochondria, which leads to the development of retinopathy. A small molecular weight G-protein essential for Nox2 activation, Rac1, is also transcriptionally activated via active DNA methylation-hydroxymethylation. DNA methylation is a dynamic process, and can also be regulated by histone modifications; diabetes alters retinal histone methylation machinery. Our aim is to investigate the role of histone methylation (H3K9me3) of *Rac1* promoter in dynamic DNA methylation- transcriptional activation. Using human retinal endothelial cells in 20 mM D-glucose, H3K9me3 at *Rac1* promoter was quantified by chromatin-Immunoprecipitation technique. Crosstalk between H3K9me3 and DNA methylation was examined in cells transfected with siRNA of histone trimethyl-transferase, *Suv39H1,* or *Dnmt1*, exposed to high glucose. Key parameters were confirmed in retinal microvessels from streptozotocin-induced diabetic mice, with intravitreally administered *Suv39H1-*siRNA or *Dnmt1*-siRNA. Compared to cells in normal glucose, high glucose increased H3K9me3 and Suv39H1 binding at *Rac1* promoter, and *Suv39H1*-siRNA prevented glucose-induced increase 5 hydroxy methyl cytosine (5hmC) and *Rac1* mRNA. Similarly, in diabetic mice, *Suv39H1*-siRNA attenuated increase in 5hmC and *Rac1* mRNA. Thus, H3K9me3 at *Rac1* promoter assists in active DNA methylation-hydroxymethylation, activating *Rac1* transcription. Regulation of Suv39H1-H3K9 trimethylation could prevent further epigenetic modifications, and prevent diabetic retinopathy.

## Introduction

The etiology of diabetic retinopathy, a slow progressing complication, is complex, and several metabolic abnormalities are implicated in its development^1,2^. Production of both cytosolic and mitochondrial reactive oxygen species (ROS) is elevated in diabetes, and increased oxidative stress is considered as one of the major metabolic abnormalities associated with the development of diabetic retinopathy^2^. We have shown that diabetes-induced increase in cytosolic ROS in the retina and its vasculature is an early event, which precedes mitochondrial damage and the development of histopathology^3,4^. These cytosolic ROS are mainly generated by NADPH oxidases, and its subunits Nox2, is one of the major ROS producing NADPH oxidases activated in the retina^3–7^. The multi-component Nox2 has both trans-membrane and cytosolic proteins, and the small G-protein, Ras-related C3 botulinum toxin substrate 1 (Rac1), is an integral cytosolic component for its activation^8^. In diabetes, Rac1 is functionally active, and while its binding with guanine exchange factors (GEFs) is increased and with its guanine dissociation inhibitor (GDIs) is decreased^9,10^. Activated Rac1, via Nox2-ROS, contributes to mitochondrial damage and the development of retinopathy^3,11^.

Diabetes also transcriptionally activates *Rac1*, and its gene expression is elevated in the retina and its capillary cells^12^. Evolving research has clearly shown that the expression of a gene can also be regulated by epigenetic modifications, without altering their DNA sequences, and epigenetic modifications are now implicated in many chronic diseases including diabetes and its complications^13–15^. Diabetic environment alters the activities of enzymes associated with epigenetic modifications, and many genes important in the development of diabetic retinopathy undergo alterations in DNA methylation, and histone acetylation and methylation ^16–19^. In diabetes, *Rac1* is also transcriptionally activated in the retina and its vasculature, and its promoter undergoes active DNA methylation-hydroxymethylation; while DNA methyl transferase 1 (Dnmt1) methylates cytosine forming 5 methyl cytosine (5mC), concomitant activation of Tet2 hydroxymethylates 5mC, forming 5 hydroxymethyl cytosine (5hmC). This opens up the chromatin for the binding of the transcription factor, and upregulates *Rac1* transcription^9,12^. Epigenetic modifications are complex and can be interrelated, and histone modifications and DNA methylation can have important role in controlling the expression of a gene^20^. For example, the same gene can be regulated by DNA and histone methylation; Dnmt1 recruitment at the promoter CpG sites can be facilitated by the methylation of lysine 9 of histone 3 (H3K9)^21^. The coordination of H3K9 methylation and DNA methylation in the regulation of *Rac1* transcription remains unclear.

The goal of this study is to investigate the role of H3K9 methylation in the regulation of *Rac1* promoter, and its association with DNA methylation-hydroxymethylation in *Rac1* transcriptional activation. Using human retinal endothelial cells (HRECs), we have investigated the effect of high glucose on H3K9 methylation on *Rac1* promoter. The role of H3K9 methylation in gene upregulation of *Rac1* was established employing specific siRNA of H3K9 trimethylation enzyme *Suv39H1,* a histone methyltransferase that trimethylates 'lysine 9’ of histone H3^22^. Cross-talk between DNA methylation and histone methylation was confirmed using *Dnmt1*-siRNA. Key parameters were confirmed in an in vivo model using retinal microvessels from mice, that were administered *Suv39H1-*siRNA or *Dnmt1*-siRNA (intravitreally) soon after induction of streptozotocin-induced diabetes.

## Results

**Figure 1:**
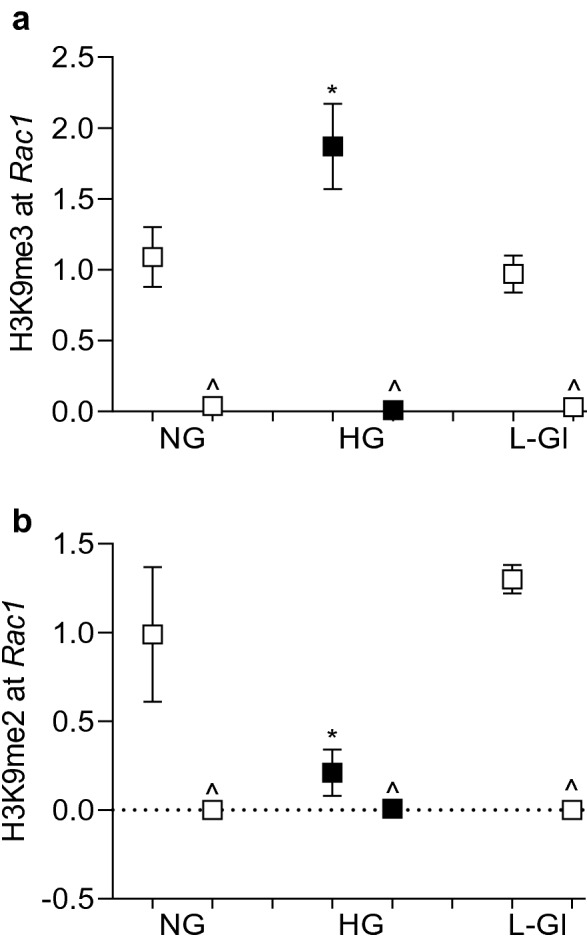
Effect of high glucose on H3K9 methylation. HRECs incubated in 5 mM (NG) or 20 mM (HG) D-glucose, or 20 mM L-glucose (L-Gl), for 96 h were analyzed for (**a**) H3K9me3 and (**b**) H3K9me2 at *Rac1* promoter by ChIP technique using normal rabbit IgG (^) as an antibody control. Each measurement was made in duplicate 3–4 different cell preparation, and the values are represented as mean ± SD. *p < 0.05 vs NG.

**Figure 2:**
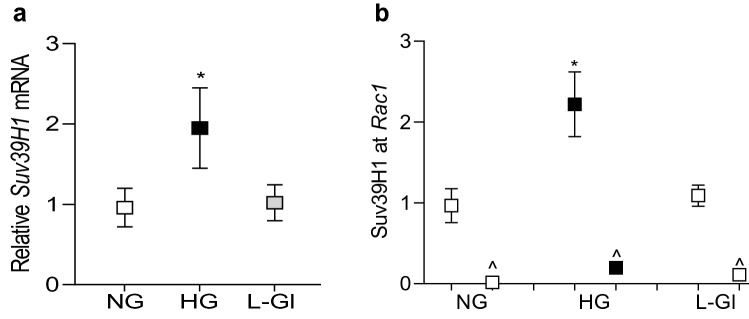
Effect of high glucose on the gene expression of *Suv39H1* and its binding at *Rac1* promoter. (**a**) *Suv39H1* gene transcripts were quantified in HRECs by qRT-PCR using β-actin as a housekeeping gene, and (**b**) binding of Suv39H1 at *Rac1* promoter was determined by ChiP technique. The values are represented as mean ± SD of measurements made in duplicate in three or more cell preparations. ^ = IgG antibody and *p < 0.05 vs NG.

Histone methylation can either enhance or repress gene transcription, and this depends on the methylation of the amino acid and the degree of methylation^23^. To investigate the role of histone methylation in transcriptional activation of *Rac1* in hyperglycemic milieu, H3K9 methylation was quantified at *Rac1* promoter, − 113 to 30 bp region, which is upstream of the Transcription Start Site. As predicted by the ‘Eukaryotic promoter database’, this region is also within the CpG island and has a predicted binding site/motif for the transcription factor Sp1^24^. Compared to cells in normal glucose, H3K9me3 levels were significantly elevated at *Rac1* promoter, but H3K9me2 levels were decreased (Fig. 1a,b). In the same cell preparations, the expression of *Suv39H1*, and its binding at the *Rac1* promoter were also significantly increased, suggesting an active trimethylation of lysine 9 of histone 3 (Fig. 2a,b). However, cells incubated in 20 mM L-glucose, instead of 20 mM D-glucose, did not show any alterations in H3K9 methylation and Suv39H1 binding at *Rac1* promoter, and the values obtained from cells in 20 mM L-glucose were not different from cells in 5 mM D-glucose, but were significantly different from those obtained from cells in 20 mM D-glucose.

**Figure 3:**
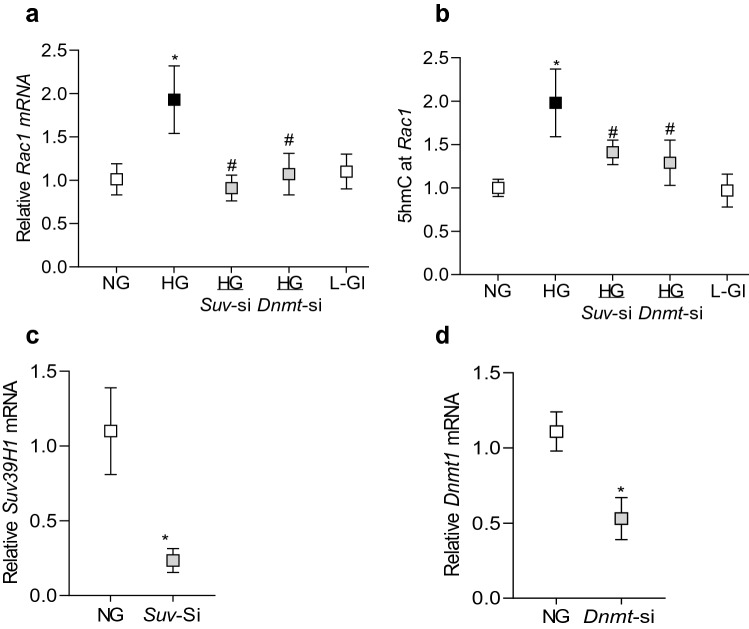
Regulation of *Rac1* gene transcripts and 5hmC at its promoter. Cells transfected with *Suv39H1*-siRNA (*Suv*-si) or *Dnmt1*-siRNA (*Dnmt*-si), incubated in high glucose for four days, were analyzed for *Rac1* (**a**) gene transcripts by qRT-PCR using β-actin as a housekeeping gene and (**b**) 5hmC at its promoter by immunoprecipitating 5hmC in the genomic DNA , and quantifying by qRT-PCR using gene specific primers. Transfection efficiency of (**c**) *SuvH39H1*-siRNAs and (**d**) *Dnmt1*-siRNA was determined by quantifying its gene transcripts. NG and HG = cells in 5 mM or 20 mM D-glucose respectively, HG/*Suv*-si and HG/*Dnmt1*-si = cells transfected with *Suv39H1*-siRNA or *Dnmt1*-siRNA, and incubated in 20 mM D-glucose, *Suv*-si and *Dnmt*-si = cells transfected with *Suv39H1*-siRNA or *Dnmt1*-siRNA. *p < 0.05 vs NG and ^#^*p* < 0.05 vs HG.

To further confirm the role of histone methylation, effect of high glucose on *Rac1* transcription was determined in cells transfected with *Suv39H1*-siRNA. As shown in Fig. 3, *Suv39H1*-siRNA attenuated glucose-induced increase in *Rac1*. Our previous work has shown that in diabetes, the binding of transcriptional factor NF-*k*B at *Rac1* promoter is increased in the retinal vasculature and due to dynamic DNA methylation-hydroxymethylation, 5hmC levels are also increased significantly^12^. To determine crosstalk between histone methylation and DNA methylation, 5hmC levels were quantified at *Rac1* promoter in the *Suv39H1*-siRNA transfected cells, exposed to high glucose. As expected, high glucose increased 5hmC by ~ twofold, and *Suv39H1*-siRNA, but not the scrambled RNA, attenuated glucose-induced increase in 5hmC, suggesting a crosstalk between histone methylation and DNA methylation (Fig. 3b). Consistent with our previous results, transfection of cells with *Dnmt1*-siRNA also attenuated glucose-induced increase in *Rac1* transcription and 5hmC levels at its promoter (Fig. 3a,b). Cells incubated in 20 mM L-glucose had similar *Rac1* expression and 5hmC at its promoter as obtained from cells in normal glucose. The transfection efficiencies of *Suv39H1*-siRNA and *Dnmt1*-siRNA are shown in Fig. 3c,d.

**Figure 4:**
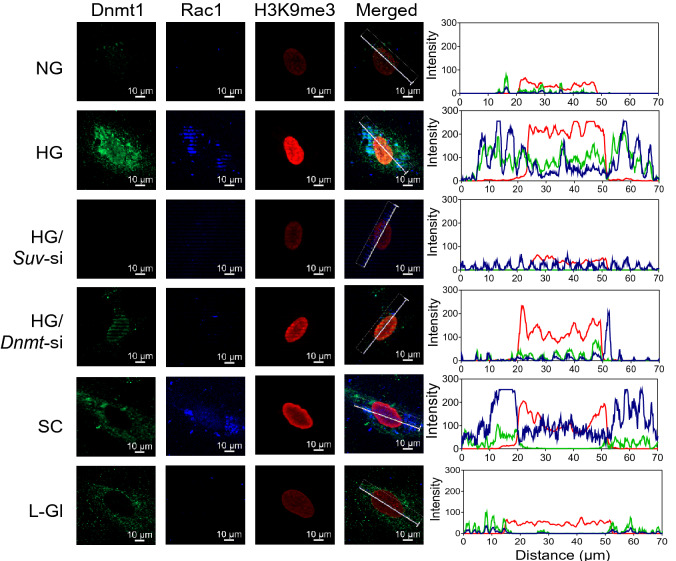
Effect of high glucose on localization of H3K9me3, Dnmt1 and Rac1. Immunofluorescence technique was used to determine co-localization of H3K9me3, Dnmt1 and Rac1 in HRECs. The images were captured by Zeiss microscope at 40 × magnification using the Apotome module. The line represents the region of interest used to quantify the arithmetic mean intensity per channel. The figure shows one representative cell/condition. Colors green, blue and red represent Dnmt1, Rac1 and H3K9me3, respectively.

Co-localization of H3K9me3, Dnmt1 and Rac1 were also confirmed by immunofluorescence technique. As shown in Fig. 4, compared to cells in normal glucose, fluorescence intensities of H3K9me3, Dnmt1 and Rac1 were significantly higher in HRECs incubated in high glucose, and *Suv39H1-*siRNA prevented increase in their intensities. However, while *Dnmt1*-siRNA ameliorated glucose-induced Dnmt1 and Rac1 intensities, it had no effect on H3K9me3 intensity, suggesting that H3K9 trimethylation makes a mark on the chromatin for DNA methylation, but DNA methylation does not facilitate H3K9 trimethylation. The accompanying line intensity profile confirms glucose-induced increased H3K9me3, Dnmt1 and Rac1 in high glucose, and the effect of *Suv39H1-*siRNA and *Dnmt1*-siRNA on their interactions.

**Figure 5:**
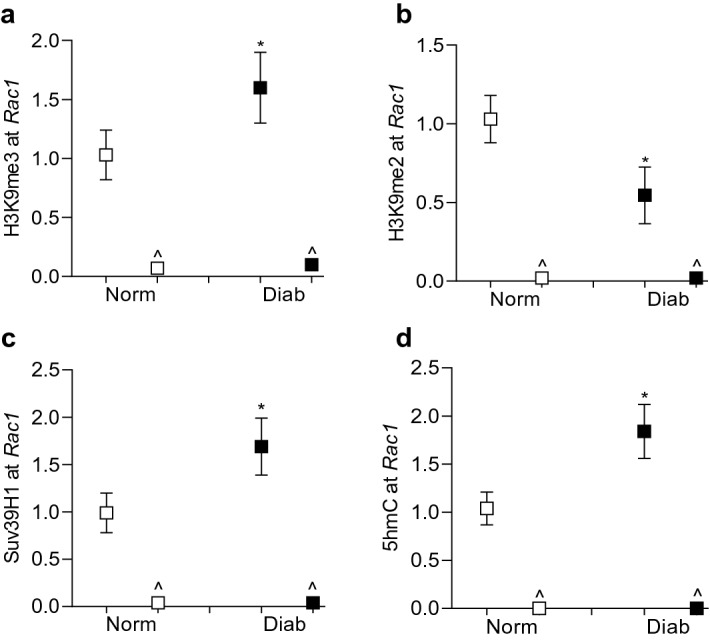
Effect of diabetes on *Rac1* promoter histone modifications in mouse retinal microvessels. Retinal microvessels from Stz-induced diabetic mice (Diab) and their age-matched normal mice (Norm) were analyzed for (**a**) H3K9me3, (**b**) H3K9me2 and (**c**) Suv39H1 at *Rac1* promoter by ChIP technique. (**d**) 5hmC levels at *Rac1* promoter were quantified by immunoprecipitating 5hmC in the genomic DNA. Normal rabbit IgG (^) was used as an antibody control. Each measurement was made in duplicate in 5–7 mice in each group. *p < 0.05 compared to normal.

**Figure 6:**
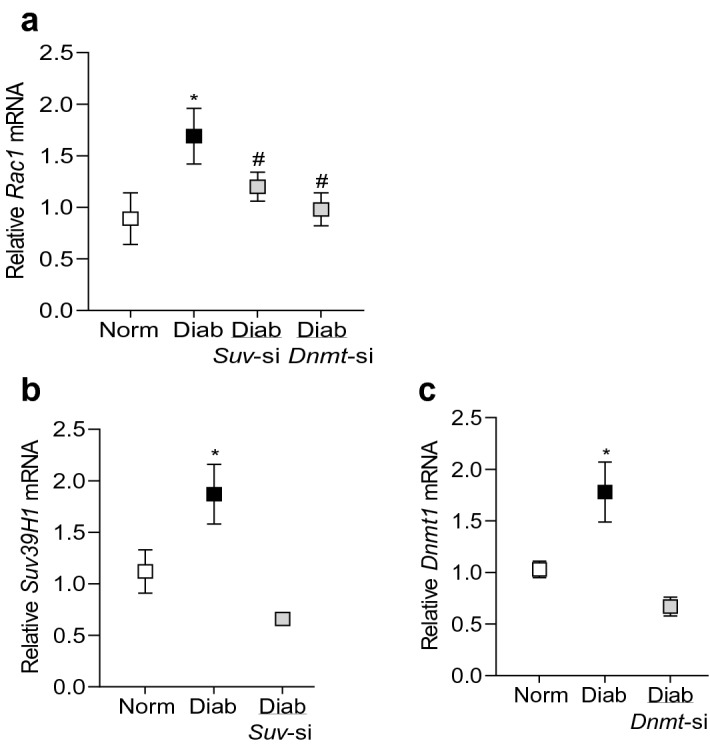
Gene expressions of *Rac1, Suv39H1* and *Dnmt1* in mouse retinal microvessels. *Rac1, Suv39H1* and *Dnmt1* gene transcripts were quantified by qRT-PCR using 18S as a housekeeping gene. The values are represented as mean ± SD of 5–6 mice/group, with each measurement made in duplicate. Norm and Diab = Normal and Diabetic mice; Diab/*Suv*-si and Diab/*Dnmt1*-si = Mice receiving *Suv39H1*-siRNA or *Dnmt1*-siRNA, soon after induction of diabetes. *p < 0.05 compared to normal and ^#^*p* < 0.05 compared to diabetes.

In accordance with the in vitro results, compared to age-matched normal mice, retinal microvasculature from diabetic mice had 80% increase in H3K9me3 levels at *Rac1* promoter, however, H3K9me2 levels were significantly decreased (Fig. 5a,b). In the same diabetic mice, *Suv39H1* binding at the promoter wasalso increased by over 70%, and 5hmC levels were elevated by ~ 75% (Fig. 5c–d). Regulation of *Suv39H1* by its siRNA prevented diabetes-induced upregulation of *Rac1* transcription; *Rac1* gene transcripts in diabetic mice receiving *Suv39H1*-siRNA were not significantly different from those obtained from normal mice (Fig. 6a). Consistent with *Suv39H1*-siRNA, as expected, *Dnmt1*-siRNA also ameliorated diabetes-induced increase in *Rac1*. Figure 6b,c show attenuation of *Suv39H1* and *Dnmt1* gene transcripts in the retinal microvessels from diabetic mice receiving their respective siRNAs.

## Discussion

Rac1 is an obligatory small molecular weight GTPase for Nox2 activation, and its membrane translocation and functional activation are regulated by many different regulators^25–27^. In the pathogenesis of diabetic retinopathy, Rac1 is activated and its expression is upregulated, and activation of Rac1-Nox2-ROS is an early event, which precedes mitochondrial damage-histopathology characteristic of diabetic retinopathy. Interactions of *Rac1* with several of its regulators, including GEFs and GDIs, are altered, and Rac1 is post-transcriptionally modified^3,10,28^. In addition, diabetes also facilitates retinal *Rac1* to undergo dynamic DNA methylation-hydroxymethylation, resulting in its transcriptional activation^12^. Histone modification machinery is altered in diabetic retinopathy, and several genes implicated in its development undergo altered histone methylation and acetylation^16,29,30^. Both histone methylation and DNA methylation can affect expression of the same gene^21,31^. Here, we show that in hyperglycemic milieu, *Rac1* promoter, in addition to undergoing dynamic DNA methylation, also undergoes histone methylation. While H3K9 trimethylation and the binding of histone methyl-transferase, *Suv39H1*, at *Rac1* promoter are increased significantly, H3K9me2 levels are decreased. Furthermore, *Suv39H1*-siRNA ameliorates glucose-induced increase in 5hmC levels at *Rac1* promoter, and its transcription. Retinal microvasculature from diabetic mice confirms the similar phenomenon. These data suggest that increase in Suv39H1-H3K9me3 at *Rac1* promoter assists in the recruitment of Dnmt1, which, via active DNA methylation-hydroxymethylation, increases *Rac1* expression.

Epigenetic modifications are now implicated in many chronic diseases including diabetes and its complications. The machinery responsible for DNA methylation and histone methylation/acetylation is altered in the retina and its capillary cells in diabetes, and many genes important in the development of diabetic retinopathy undergo epigenetic modifications including DNA methylation and histone acetylation and methylation^18,19^. Our recent work has shown that while activation of Dnmt1 methylates cytosines at *Rac1* promoter, concomitant activation of Tet2 converts 5mC to 5hmC, resulting in its transcriptional activation^17^. Here, we show that *Rac1* promoter also undergoes histone methylation, while the levels of H3K9me2 are decreased, that of H3K9me3 are increased, and the binding of Suv39H1 is increased. Although Suv39H1 catalyzes both di- and tri- methylation of H3K9 by preferentially binding to H3K9me1^32^, our data clearly suggest that its binding at *Rac1* promoter rapidly converts H3K9me2 formed into H3K9me3, resulting in decreased H3K9me2 and increased H3K9me3 . However, the role of the JmjC-containing lysine demethylase, KDM4D^33^, or any other demethylase, in demethylation of H3K9me3 to H3K9me2 cannot be ruled out.

Regulation of histone methylation enzyme, *Suv39H1,* by its specific siRNA inhibits hyperglycemia-induced increase in 5hmC at *Rac1* promoter and ameliorates increase in *Rac1* gene transcripts, implying a close cross-talk between DNA methylation and H3K9 methylation. In support, both genomic and biochemical data have shown a strong association between DNA methylation and H3K9 methylation, and in *Suv39H1* knockout mice, DNA methylation is lost^20^.

As mentioned above, SUV39H1 is responsible for trimethylation at H3K9 to H3K9me3^34^; our results show that its expression and binding at *Rac1* promoter are increased in hyperglycemia, and silencing of *Suv39H1* attenuates increase in 5hmC, which is a product of dynamic DNA methylation. This suggests the possibility that Suv39H1 binding creates a mark, which facilitates Dnmt1 binding. In support, Suv39H1-mediated H3K9 methylation is shown to direct DNA methylation^35^, and Dnmt1 is considered necessary to maintain trimethylation of H3K9 at pericentromeric regions in human cancer cells^36^. *Suv39H1*-siRNA also attenuates hyperglycemia-induced increase in *Rac1* expression, further supporting the role of H3K9me3 at *Rac1* promoter in DNA methylation. We recognize that our in vitro model employed only retinal endothelial cells, however, in the pathogenesis of diabetic retinopathy retinal pericytes also undergo accelerated apoptosis^37^, and we cannot rule out similar glucose-induced epigenetic changes in *Rac1* promoter in retinal pericytes.

The results obtained from in vitro model, retinal endothelial cells in high glucose, are duplicated in in vivo model; retinal microvessels from diabetic mice also have increased *Rac1* transcription, and increased SuvH39H1 and H3K9me3 and decreased H3Kme2 at *Rac1* promoter. Furthermore, regulation of histone trimethylation or DNA methylation-hydroxymethylation by intravitreal administration of *SuvH39H1*-siRNA or *Dnmt1*-siRNA, soon after induction of diabetes, prevents increase in *Rac1* gene transcripts, further supporting a close cross-talk between histone methylation and DNA methylation in regulating *Rac1* transcription.

Our study is focused on crosstalk between H3K9 methylation and DNA methylation-hydroxymethylation in *Rac1* transcriptional activation in diabetes, however, we recognize that methylation of lysine, other than K9 of histone 3, could be helping in recruiting Dnmt1 and increasing *Rac1* transcription; activation of an enzyme responsible for methylation of lysine 27 of histone 3, Enhancer of Zeste homolog 2 (Ezh2), in diabetes is also associated with active DNA methylation and transcriptional activation of retinal *MMP-9*^38^, and role of Ezh2 in *Rac1* transcriptional activation remains a possibility. Furthermore, the role of other histone methyl- transferases or -demethylases, and histone acetylation, which is also affected in diabetes^29^, in *Rac1* transcription, cannot be ruled out.

The results presented here are from in vitro and in vivo models of diabetic retinopathy, and the results from these two models complement each other. To transition to the human disease, we have shown similar increase in Rac1 functional activation in retinal microvasculature from human donors with documented diabetic retinopathy, compared to their age-matched nondiabetic donors^3,9^. In addition, the microvasculature from donors with documented diabetic retinopathy also presents comparable activation of the DNA methylation-hydoxymethylation enzymatic machinery^39^ and histone modifications^16^, as seen in the in vivo and in vitro models of diabetic retinopathy. Thus, there remains a strong possibility of similar epigenetic modifications of *Rac1* promoter in the retinal microvasculature from human donors with diabetic retinopathy, which would further strengthen the clinical significance of the results presented here.

**Figure 7:**
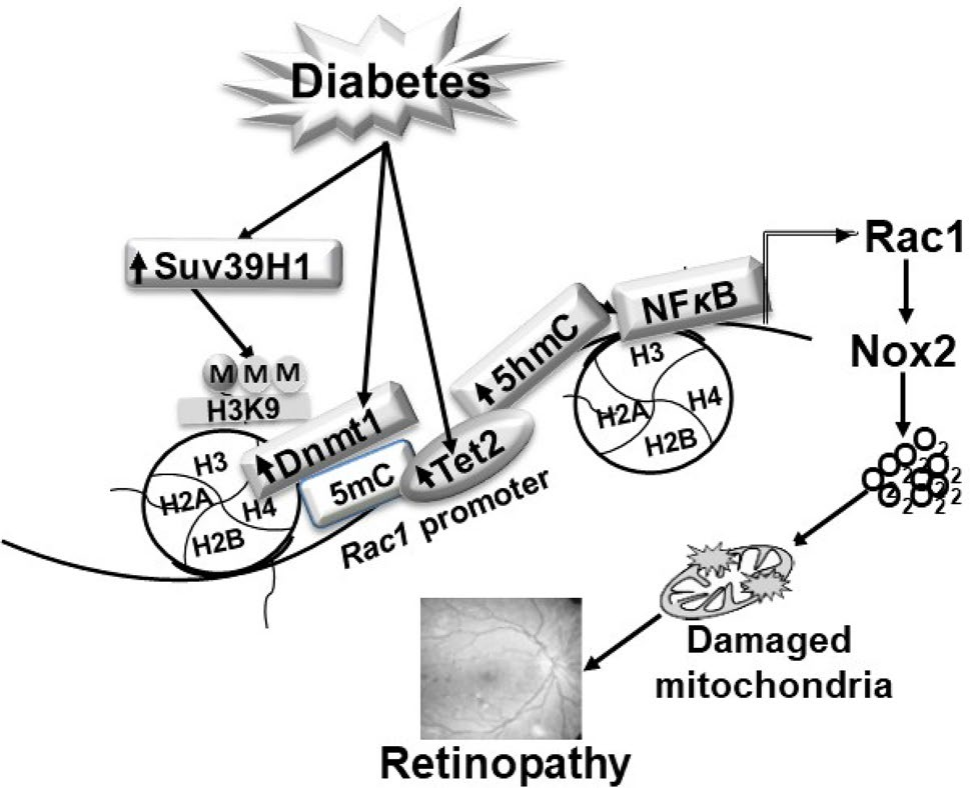
Working model: Diabetes activates Suv39H1, and this increases H3K9me3 at *Rac1* promoter, creating a mark on the chromatin for Dnmt1 binding. Concomitant activation of Dnmts and Tets hydroxymethylates 5mC to 5 hmC, facilitating the binding of the transcription factor and induction of *Rac1* transcription. Activation of Rac1-Nox2-ROS damages the mitochondria, ultimately leading to the development of diabetic retinopathy.

Thus, using both in vitro and in vivo models of diabetic retinopathy, our results provide strong evidence of relationship between H3K9 methylation and DNA modifications of *Rac1* promoter in its transcriptional activation. We demonstrate that diabetes increases the recruitment of Suv39H1 at *Rac1* promoter, which assists in the recruitment of Dnmt1, initiating an active DNA methylation-hydroxymethylation and transcriptional activation (Fig. 7). Regulation of *Suv39H1*, in addition to protecting H3K9 trimethylation, also protects active *Rac1* promoter DNA methylation-hydroxymethlation. Activation of Rac1 is an early event in the pathogenies of diabetic retinopathy, and sustained activation of Rac1-Nox2-ROS damages the mitochondria, initiating a self-propagating a vicious cycle of free radicals. In the later stages of diabetic retinopathy, via regulating angiogenic signaling pathways of VEGF and hypoxia-inducible factors, Rac1 could contribute to the neovascularization^40^. In addition, Rac1 activation also contributes to platelet aggregation by affecting platelet actin cytoskeleton rearrangements^41^. Thus, regulation of *Rac1* gene expression by targeting the machinery responsible for its epigenetic modifications, e.g., Suv39H1, could prevent both histone and DNA methylation, ameliorating abnormalities in vascular and platelet dysfunction associated with diabetic retinopathy, and ultimately, slowing down/halting its progression.

## Methods

### Retinal endothelial cells

HRECs, obtained from Cell Systems Corporation (Cat. No. ACBRI 181, Cell Systems Corp, Kirkland, WA, USA), were cultured in Dulbecco’s modified Eagle medium (DMEM) containing 12% heat-inactivated fetal bovine serum, endothelial cell growth supplement (15 µg/ml), insulin/transferrin/selenium (1%), Glutamax (1%) and antibiotic/antimycotic (1%) in an environment of 95% O_2_ and 5% CO_2,_ as described previously^42,43^. Cells from 6th–9th passage were incubated in 5 mM (NG) or 20 mM (HG) D-glucose for 96 h. Parallel osmotic/metabolic control included HRECs incubated in 20 mM L-glucose.

A batch of HRECs from 6th-7th passage were transfected with two independent siRNA pools of *Suv39H1* (Cat No: AM4611, siRNA ID: 117147; 117149, Thermofisher Scientific, Waltham, MA, USA) using Lipofectamine™ RNAiMAX Transfection Reagent (Cat. No. 13778150, Invitrogen™, Carlsbad, CA, USA)^42^, or with *Dnmt1-*siRNA (Cat. No. SC-35204, Santa Cruz Biotechnology, Santa Cruz, CA, USA), using their transfection reagent (Cat. No. SC-29528, Santa Cruz Biotechnology). The transfected cells were incubated in 5 mM or 20 mM D-glucose for 96 h. The transfection efficiency was evaluated by quantifying mRNA levels by SYBR green-based quantitative real time PCR (qRT-PCR). While cells transfected with *Suv39H1*-siRNA ID: 117147, had ~ 75% reduction in its mRNA, cells transfected with ID 117149 had less than 40% reduction; *Suv39H1*-siRNA ID: 117147 was used in the subsequent experiments.

### Mouse

Seven to eight weeks old C57BL/6 J mice (either sex) were made diabetic by streptozotocin injection (55 mg/kg BW for 4 consecutive days). Mice with blood glucose over 250 mg/dl two days after the last injection were considered diabetic. Soon after establishment of diabetes, mice were divided in four different groups, while mice in group 1 remained diabetic, mice in groups 2 and 3 received intravitreal administration of two independent *Suv39H1*-siRNA pools (ID: MSS238068 and MSS277491 respectively, Thermofisher Scientific), and mice in group 4 received *Dnmt1*-siRNA (ID: MSS203624; Thermo Fisher Scientific)^12^. Briefly, 2 µg siRNA was suspended in 2 µl nuclease free water and mixed with Invivofectamine (Cat. No. IVF 3001, Invitrogen, Carlsbad, CA, USA). The right eye received siRNA and the left eye received a medium GC content negative control siRNA (Cat. No. 12935-300, Thermo Fisher Scientific), as reported previously^12,44^. Four weeks after administration of siRNA, the mice were sacrificed, and the retina was quickly isolated. Age-matched normal mice and diabetic mice, without any siRNA, served their controls. The treatment on animals is in accordance with the Association for Research in Vision and Ophthalmology Resolution on the Use of Animals in Research. The experimental protocols followed were approved by Wayne State University’s Animal Care and Use Committee, and all methods are reported in accordance with ‘Animal Research Reporting of In Vivo Experiments (ARRIVE)’ guidelines.

Retinal microvessels were prepared by incubating the retina in 5 ml de-ionized water for 60 min in a shaking water bath at 37 °C. The nonvascular tissue was gently removed under the microscope^9,12^.

Chromatin immunoprecipitation (ChIP): H3K9 methylation and the binding of *Suv39H1* and Dnmt1 at the *Rac1* promoter was determined by ChIP assay using cross-linked samples sonicated in ChIP lysis buffer. Protein-DNA complex (100 µg) was immunoprecipitated with antibodies (3 µg each) against either *Suv39H1* (Cat No.05-615, Millipore, Billerica, MA, USA) or H3K9me2, H3K9me3 or Dnmt1 (Cat No. ab1220, ab8898 and ab13537, respectively, Abcam, Cambridge, MA, USA). Each experiment included an antibody control, where the samples were immunoprecipitated with IgG (Cat. No. ab171870, Abcam). The immunoprecipitated complex was captured using Protein A Agarose/Salmon Sperm DNA (Cat. No. 16157, EMD Millipore, Temecula, CA, USA), washed and de-crosslinked at 65 °C for 6 h, followed by DNA isolation with phenol: choloroform: isoamylalcohol, using the methods reported previously^12,16,42^. Relative abundance of the target at the promoter was quantified by qRT-PCR using *Rac1* promoter specific primer (Table 1). The target values were normalized to the input controls to obtain fold change.

**Table 1:** Primer sequences.

Gene	Sequence (5′–3′)
**Human**	
*Rac1* promoter (-113 to 30)	CTTCCGAGCATTCCCGAAGTC
	AATGGCCGCTCCACTCAC
*Rac1*	CGCCCCCTATCCTATCCGCA GAACACATCGGCAATCGGCTTGT
*Suv39H1*	ATGGCTGTACGTGGTTACGG
	CTGCAGCCTGTCAGTGGG
*Dnmt1*	AGTCCGATGGAGAGGCTAAG
	TCCTGAGGTTTCCGTTTGGC
*β-actin*	AGCCTCGCCTTTGCCGATCCG
	TCTCTTGCTCTGGGCCTCGTCG
**Mouse**	
*Rac1* promoter (− 957 to − 1061)	CGGAACCCCGTGGTCAATAA
	CCCACAAGACGACAGGGAAA
*Rac1*	CACGACCAATGCATTTCCTGG
	AAGAACACGTCTGTCTGCGG
*Suv39H1*	TGTCAACCATAGTTGTGATCC
	ATTCGGGTACTCTCCATGTC
*Dnmt1*	CCTAGTTCCGTGGCTACGAGGAGAA
	TCTCTCTCCTCTGCAGCCGACTCA
*18S*	GCCCTGTAATTGGAATGAGTCCACTT
	CTCCCCAAGATCCAACTACGAGCTTT

Gene transcripts were quantified by qRT-PCR using gene/species-specific primers (Table 1), and the specific products were confirmed by SYBR green single melt curve analysis. The results were normalized to the expression of the housekeeping gene *β-actin* (human) *or 18S* (mice), and the relative fold change was calculated using delta delta Ct method^12,44^.

Quantification of 5hmC: Genomic DNA was isolated using Qiagen DNA isolation kit (Qiagen, Valencia, CA, USA), and immunoprecipitated with 5hmC antibody. The level of 5hmC was quantified using hydroxymethylated DNA Immunoprecipitation (hMeDIP) kit (Cat. No. P-1038, EPIGENTEK, Farmingdale, NY, USA). The enrichment of 5hmC at the *Rac1* promoter was analyzed by qRT-PCR using specific primers^12^.

Localization of H3K9me3, Dnmt1, and Rac1 was performed by immunofluorescence technique using unlabeled H3K9me3 rabbit primary antibody (Cat. no. ab8898), DyLight® 488 (Cat. no. ab201799) conjugated Dnmt1 (Cat. no. ab13537) mouse primary antibody, and DyLight® 350 (Cat. no. ab201797) conjugated Rac1 (Cat. no. ab33186) mouse primary antibody, each antibody at 1:100 dilution. Texas red-conjugated anti-rabbit (1:100 dilution) secondary antibody was used to counter stain H3K9me3. The coverslips were mounted on using Vectashield mounting medium (Cat. no. H-1000, Vector Laboratories, Burlingame, CA, USA), and images were captured by Zeiss microscope at 40 × magnification using the Apotome module. The line represents the region of interest used to quantify the arithmetic mean intensity per channel using Zeiss software module.

### Statistical analysis

Results are presented as mean ± SD, and group comparison were made using one-way ANOVA followed by Dunn’s test, and a P value less than 0.05 was considered significant.

## References

[1] Frank RN (2004). Diabetic retinopathy. N. Engl. J. Med..

[2] Kowluru RA, Mishra M (2015). Oxidative stress, mitochondrial damage and diabetic retinopathy. Biochim. Biophys. Acta.

[3] Kowluru RA, Kowluru A, Veluthakal R (2014). TIAM1-RAC1 signalling axis-mediated activation of NADPH oxidase-2 initiates mitochondrial damage in the development of diabetic retinopathy. Diabetologia.

[4] Kumar B, Kowluru A, Kowluru RA (2015). Lipotoxicity augments glucotoxicity-induced mitochondrial damage in the development of diabetic retinopathy. Invest. Ophthalmol. Vis. Sci..

[5] Frey RS, Ushio-Fukai M, Malik AB (2009). NADPH oxidase-dependent signaling in endothelial cells: Role in physiology and pathophysiology. Antiox Redox Signal.

[6] Henríquez-Olguín C, Boronat S, Cabello-Verrugio C, Jaimovich E, Hidalgo E, Jensen TE (2019). The emerging roles of nicotinamide adenine dinucleotide phosphate oxidase 2 in skeletal muscle redox signaling and metabolism. Antiox Redox Signal.

[7] Al-Shabrawey M, Rojas M, Sanders T (2008). Role of NADPH oxidase in retinal vascular inflammation. Invest. Ophthalmol. Vis. Sci..

[8] Bosco EE, Mulloy JC, Zheng Y (2009). Rac1 GTPase: A "Rac" of all trades. Cell Mol. Life Sci..

[9] Mohammad G, Duraisamy AJ, Kowluru A, Kowluru RA (2019). Functional regulation of an oxidative stress mediator, Rac1, in Diabetic Retinopathy. Mol Neurobiol.

[10] Sahajpal N, Kowluru A, Kowluru RA (2019). The regulatory role of Rac1, a small molecular weight GTPase, in the development of diabetic retinopathy. J. Clin. Med..

[11] Kowluru RA, Mishra M, Kumar B (2016). Diabetic retinopathy and transcriptional regulation of a small molecular weight G-Protein, Rac1. Exp. Eye Res..

[12] Duraisamy AJ, Mishra M, Kowluru A, Kowluru RA (2018). Epigenetics and regulation of oxidative stress in diabetic retinopathy. Invest. Ophthalmol. Vis. Sci..

[13] Zhang TY, Meaney MJ (2010). Epigenetics and the environmental regulation of the genome and its function. Annu. Rev. Psychol..

[14] Cooper ME, El-Osta A (2010). Epigenetics: Mechanisms and implications for diabetic complications. Circ. Res..

[15] Cavalli G, Heard E (2019). Advances in epigenetics link genetics to the environment and disease. Nature.

[16] Zhong Q, Kowluru RA (2013). Regulation of matrix metalloproteinase-9 by epigenetic modifications and the development of diabetic retinopathy. Diabetes.

[17] Kowluru RA, Shan Y, Mishra M (2016). Dynamic DNA methylation of matrix metalloproteinase-9 in the development of diabetic retinopathy. Lab. Invest..

[18] El-Osta A, Brasacchio D, Yao D (2008). Transient high glucose causes persistent epigenetic changes and altered gene expression during subsequent normoglycemia. J. Exp. Med..

[19] Bell CG, Teschendorff AE, Rakyan VK, Maxwell AP, Beck S, Savage DA (2010). Genome-wide DNA methylation analysis for diabetic nephropathy in type 1 diabetes mellitus. BMC Med. Genom..

[20] Du J, Johnson LM, Jacobsen SE, Patel DJ (2015). DNA methylation pathways and their crosstalk with histone methylation. Nat. Rev. Mol. Cell Biol..

[21] Estève PO, Chin HG, Benner J (2009). Regulation of DNMT1 stability through SET7-mediated lysine methylation in mammalian cells. Proc. Natl. Acad. Sci. USA.

[22] Rea S, Eisenhaber F, O'Carroll D (2000). Regulation of chromatin structure by site-specific histone H3 methyltransferases. Nature.

[23] Black JC, Van Rechem C, Whetstine JR (2012). Histone lysine methylation dynamics: Establishment, regulation, and biological impact. Mol. Cell.

[24] Dreos R, Ambrosini G, Groux R, Cavin Périer R, Bucher P (2017). The eukaryotic promoter database in its 30th year: Focus on non-vertebrate organisms. Nucleic Acids Res..

[25] Sarfstein R, Gorzalczany Y, Mizrahi A (2004). Dual role of Rac in the assembly of NADPH oxidase, tethering to the membrane and activation of p67phox: A study based on mutagenesis of p67phox-Rac1 chimeras. J. Biol. Chem..

[26] Bokoch GM, Zhao T (2006). Regulation of the phagocyte NADPH oxidase by Rac GTPase. Antiox Redox Signal.

[27] Payapilly A, Malliri A (2018). Compartmentalisation of RAC1 signalling. Curr. Opin. Cell Biol..

[28] Kowluru RA. Retinopathy in a diet-induced type 2 diabetic rat model, and role of epigenetic modifications. *Diabetes* **69**, 689-698 (2020). 10.2337/db19-1009 PMC7085254 31949005

[29] Zhong Q, Kowluru RA (2010). Role of histone acetylation in the development of diabetic retinopathy and the metabolic memory phenomenon. J. Cell Biochem..

[30] Zhong Q, Kowluru RA (2011). Epigenetic changes in mitochondrial superoxide dismutase in the retina and the development of diabetic retinopathy. Diabetes.

[31] Stoll S, Wang C, Qiu H (2018). DNA methylation and histone modification in hypertension. Int. J. Mol. Sci..

[32] Rao VK, Pal A, Taneja R (2017). A drive in SUVs: From development to disease. Epigenetics.

[33] Zoabi M, Nadar-Ponniah PT, Khoury-Haddad H (2014). RNA-dependent chromatin localization of KDM4D lysine demethylase promotes H3K9me3 demethylation. Nucleic Acids Res..

[34] Blusztajn JK, Mellott TJ (2012). Choline nutrition programs brain development via DNA and histone methylation. Cent. Nerv. Syst. Agents Med. Chem..

[35] Lehnertz B, Ueda Y, Derijck AA (2003). Suv39h-mediated histone H3 lysine 9 methylation directs DNA methylation to major satellite repeats at pericentric heterochromatin. Curr. Biol..

[36] Espada J, Ballestar E, Fraga MF (2004). Human DNA methyltransferase 1 is required for maintenance of the histone H3 modification pattern. J. Biol. Chem..

[37] Mizutani M, Kern TS, Lorenzi M (1996). Accelerated death of retinal microvascular cells in human and experimental diabetic retinopathy. J. Clin. Invest..

[38] Duraisamy AJ, Mishra M, Kowluru RA (2017). Crosstalk between histone and DNA methylation in regulation of retinal matrix metalloproteinase-9 in diabetes. Invest. Ophthalmol. Vis. Sci..

[39] Mishra M, Kowluru RA (2015). Epigenetic modification of mitochondrial DNA in the development of diabetic retinopathy. Invest. Ophthalmol. Vis. Sci..

[40] Ushio-Fukai M, Alexander RW (2004). Reactive oxygen species as mediators of angiogenesis signaling: Role of NAD(P)H oxidase. Mol. Cell Biochem..

[41] Schiattarella GG, Carrizzo A, Ilardi F (2018). Rac1 modulates endothelial function and platelet aggregation in diabetes mellitus. J. Am. Heart Assoc..

[42] Duraisamy AJ, Mohammad G, Kowluru RA (2019). Mitochondrial fusion and maintenance of mitochondrial homeostasis in diabetic retinopathy. Biochim. Biophys. Acta Mol. Basis Dis..

[43] Radhakrishnan R, Kowluru RA (2021). Long noncoding RNA MALAT1 and regulation of the antioxidant defense system in diabetic retinopathy. Diabetes.

[44] Kowluru RA, Mohammad G (2020). Epigenetics and mitochondrial stability in the metabolic memory phenomenon associated with continued progression of diabetic retinopathy. Sci. Rep..

